# High intensity functional training for people with spinal cord injury & their care partners

**DOI:** 10.1038/s41393-024-00977-8

**Published:** 2024-03-22

**Authors:** Reed Handlery, Kaci Handlery, Dana Kahl, Lyndsie Koon, Elizabeth W. Regan

**Affiliations:** 1 Arkansas Colleges of Health Education, School of Physical Therapy, 7006 Chad Colley Blvd, Fort Smith, AR 72916 USA; 2 Research and Training Center on Independent Living, University of Kansas, Lawrence, KS USA; 3 University of South Carolina, Department of Exercise Science, Physical Therapy Program, Columbia, SC USA

**Keywords:** Public health, Spinal cord diseases

## Abstract

**Study design:**

Non-randomized clinical trial.

**Objectives:**

Examine the feasibility, physical and psychosocial effects of a high intensity functional training (HIFT) exercise program for people with spinal cord injury (pSCI) and their care partners (CPs).

**Setting:**

Community fitness center in a Medically Underserved Area (Fort Smith, USA.)

**Methods:**

A single-group design with three assessment points (before the program, at midpoint (13 weeks), and post-program (25 weeks) was used to examine the effects of up to 49 HIFT sessions over 25-weeks. Sessions were 60 to 75 min in duration and adapted to the abilities of participants. Feasibility measures included recruitment, retention, attendance, safety and fidelity (exercise intensity rated via session-Rating of Perceived Exertion (RPE). Physical measures included cardiovascular endurance, anaerobic power, and muscular strength. Psychosocial measures included perceived social support for exercise, exercise self-efficacy and health-related quality of life.

**Results:**

Fourteen pSCI (7 with paraplegia and 7 with tetraplegia, 2 females) and 6 CPs (4 females) were included (median age = 60) (IQR = 15.8). Recruitment rates were 40% for pSCI and 32% for CPs. On average, participants attended 73% (22%) of exercise sessions with a median session-RPE of 5 (IQR = 1). Retention rates were 83% and 67% for pSCI and CPs, respectively. For pSCI and their CPs, large effect sizes were observed for cardiovascular endurance, anaerobic power, muscular strength, and social support for exercise.

**Conclusions:**

For pSCI and their CPs, HIFT appears feasible and potentially leads to improvements in physical and psychosocial health for both groups.

## Introduction

Exercise is highly beneficial for people with and without spinal cord injury (SCI). General benefits of exercise include improved cardiovascular endurance, muscular strength, and reduced risk of developing or worsening cardiovascular disease, certain cancers and type 2 diabetes [1]. Specific benefits for people with SCI (pSCI) include improved cardiovascular endurance, muscular strength, quality of life, exercise self-efficacy, fatigue, stress, and depression [2–4]. Unfortunately, sixty five percent of pSCI in the United States do not exercise enough to obtain noticeable health benefits [5]. This lack of exercise is detrimental not only to pSCI but potentially to their care partners (CPs), as their health is often linked [6]. For example, if a person with SCI experiences a cardiovascular event, this could lead to increased reliance on the CP for daily tasks like mobility and self-care, thus increasing CP burden. If a CP experiences increased burden, they are less likely to engage in exercise [7]. This is important because CPs also exhibit higher levels of depression [8] and have increased odds of developing cardiovascular disease [9]; both of which can be mitigated with exercise. Additionally, there is a public health recommendation to promote exercise at multiple levels of the socio-ecological model (e.g., individual, interpersonal, community) [10, 11], rather than solely focusing on pSCI. Thus, incorporating both pSCI and CPs in exercise interventions may lead to a larger impact on public health through increases in exercise performance.

High intensity functional training (HIFT) is an exercise program that uses various functional movements performed at a high-intensity to improve general fitness and performance [12]. In contrast to high intensity interval training (HIIT), which typically involves a single, often aerobic exercise mode (e.g., cycling), HIFT combines both aerobic and anaerobic (e.g., weightlifting) exercises, specifically targeting important movements needed for pSCI (e.g., wheelchair skills and mobility, transfers, lifting objects from the floor, lifting objects overhead, and carrying objects). Consequently, improvements in muscle strength and power can occur alongside improvements in cardiovascular endurance [13]. In 2023, HIFT was rated the sixth most popular fitness trend in the U.S., outranking HIIT by one spot [14]. Performing HIFT in a group may also be superior to traditional moderate-intensity aerobic and resistance training in terms of participant enjoyment and intentions to continue exercising [15], an important finding given the prevalence of physical inactivity in pSCI.

While HIFT is extremely popular in the general population [16] and pSCI are already participating in HIFT [17], there has been no research to date examining HIFT in pSCI nor CPs.

This evidence gap is significant because CrossFit, the most common form of HIFT, is implemented in over 15,000 exercise facilities worldwide [16]. Thus, HIFT programs could serve as an abundantly available opportunity to help pSCI and their CPs improve their lives through fitness.

The present study utilized a 25-week HIFT program which had several innovative attributes, (1) CPs were active participants, (2) the program consisted of over a hundred different exercises (some of which never reported in SCI literature) that were adapted to meet the needs of a wide range of pSCI and CPs, (3) select workouts required teamwork to complete, and (4) non-traditional exercise parameters (e.g., completing as many repetitions as possible in a set amount of time) were utilized. The purpose of this study was to examine the feasibility of a 25-week HIFT program for pSCI and their CPs. Second, we explored the effects of HIFT on physical and psychosocial outcomes for both groups.

## Methods

### Study design & participants

We used a single-group design with assessments occurring at three time points (before the program (T1)), at midpoint (13 weeks, T2) and post-program (25-weeks, T3). Rolling admission was used, where prospective participants could begin the study at any time; thus, not all participants participated in all 25 weeks of the program.

Inclusion criteria for pSCI included age 18 years or older, a self-reported diagnosis of SCI with an injury level of C5 (ASIA A-D) or below and/or at least 4/5 elbow flexion strength bilaterally, ability to ambulate and/or propel a power or manual wheelchair independently, ability to communicate and read in English, and ability to provide transport to and from the fitness facility. Exclusion criteria for pSCI included failure to obtain medical clearance to exercise at a high intensity from a physician. Inclusion and exclusion criteria for CPs were identical to those with SCI other than SCI diagnosis.

Participants were recruited through local healthcare providers, social media, and word of mouth from enrolled participants. The program was free for participants. They received a t-shirt and water bottle for participating but no other compensation was provided. As the primary aim of this study was assessing feasibility, no power analysis was conducted. This study received approval from the Arkansas Colleges of Health Education’s Institutional Review Board (PT-2021-024) and was prospectively registered on ClinicalTrials.gov (NCT05221723). All participants provided written informed consent prior to any data collection.

### HIFT program

#### Coaches

There were two primary coaches, both licensed physical therapists. One coach with a Level 1 CrossFit certification, designed and led all exercise sessions. The other primary coach was a Board-Certified Clinical Specialist in Neurologic Physical Therapy (NCS). Intermittently, additional support was provided by another physical therapist with NCS, first year physical therapist students and/or first year osteopathic medicine students.

#### Facility & Equipment

Exercise sessions were held at CrossFit Fort Smith, an 8000 square-foot facility with accessible restrooms, parking, and floorplan. The facility was heated, but the only forms of cooling were two large ceiling fans and two large garage doors.

The facility contained common CrossFit equipment including squat racks, air-bikes, row ergometers, medicine balls and free weights. To increase exercise options we were able to add grip aids, lap belts, ski ergometers, bike ergometers, various ropes, lighter free weights, indoor sleds, and boxing equipment to the facility.

### Exercise sessions

With one exception, group exercise sessions were held twice weekly on Tuesdays and Thursdays and were 60–75 min in duration. There were 49 sessions across 25 weeks, with a 5-week break between sessions 25 and 26. This break was required in July for safety as facility temperatures exceeded 32 °C (90 °F). Sessions were designed to meet or exceed SCI-specific exercise guidelines [18].

In general, sessions began with a 10-min structured warmup consisting of 3–5 min of participant-chosen aerobic activity, followed by a series of exercises designed to improve shoulder stability (Supplementary Appendix 1). Following the warmup, 20–60 min were spent on the workout of the day (WOD), which emphasized multi-modal, functional movements performed at a high intensity. Occasionally, a general cooldown consisting of lighter intensity exercise was performed for 5 min or less at the end of sessions, though sometimes the cooldown was passive rest.

Traditional exercise programs for pSCI prescribe a certain number of sets and repetitions for resistance exercises or a specific time for aerobic exercises. Conversely, the current study predominantly used HIFT methods common in CrossFit. These include As Many Repetitions (or rounds) As Possible (AMRAP): participants perform a series of exercises as many times as they can within a specific time frame, Every Minute On The Minute (EMOM): participants perform a certain number of repetitions for 1, 1.5, 2..etc. minutes, and when they complete the repetitions, they rest for the remainder of the time, and Repetitions (or rounds) for Time (RFT): participants complete a certain number of repetitions as fast as able [12]. Other methods included partner or team WODs: multiple participants work together to complete the prescribed exercise(s), interval training: participants exercise and rest for defined periods, and traditional training: participants perform a specific number of sets and repetitions or perform aerobic activity for a certain time or distance. The content for all exercise sessions is provided in Supplementary Appendix 2, including various exercise versions to accommodate participants with paraplegia, tetraplegia and those who were ambulatory.

To acclimate participants, the first four weeks of the program were prescribed with lower volumes (e.g., shorter AMRAPs) and subsequently increased in later sessions. Coaches provided extra guidance for participants who joined the program after the first four weeks, including monitoring exertion, helping choose appropriate intensity of exercise to ensure safety, and encouraging participants to “start low and go slow” as they became acclimated to the program. As is customary with HIFT performed in the community setting, participants were responsible for self-regulating their performance and intensity during exercise. Coaches pragmatically progressed or regressed exercises to ensure appropriate safety and intensity (e.g., increasing a weight during the start of a WOD and decreasing it later). Coaches progressed exercises for participants when the reported Rate of Perceived Exertion (RPE) was <5 out of 10 or when the participant was able to perform an exercise without any difficulty (e.g., no rest required or no change in movement speed).

### Adapting & tailoring

All CPs actively participated in the exercise program. They did not physically assist pSCI as the focus of the program was HIFT for both pSCI and CPs. Occasionally during partner or team WODs, pSCI and their CPs were paired but more commonly, participants were paired by coaches based on ability levels and personalities. Because CrossFit was designed for able-bodied adults and the majority of our participants utilized wheelchairs, we did not prescribe many of CrossFit’s foundational movements (e.g., squats, push press, push jerk) and had to adapt others (e.g., deadlift, shoulder press, medicine ball clean). However, we prescribed exercises that targeted CrossFit’s ten physical skills (cardiovascular endurance, strength, flexibility, power, speed, coordination, agility, balance, accuracy, stamina), though cardiovascular endurance and muscular strength were prioritized based on SCI-exercise guidelines [18] (Supplementary Appendix 2).

Exercise programming was first tailored to manual wheelchair users and then adapted to meet the needs of powerchair users or ambulators (including all CPs) (Supplementary Appendix 2). Adapting was frequently used to ensure all participants’ safety, efficacy, and inclusion. For example, if the WOD prescribed 5 hoists, 10 medicine ball wall balls (arms only), and 15 medicine ball rotations for a manual wheelchair user, ambulators could instead perform 5 hoists, 10 medicine ball wall balls (added squat prior to throwing ball) and 15 medicine ball rotations. In contrast, powerchair users could perform 5 hoist holds (rather than grasping and releasing to hoist the weight), 10 medicine ball front raises (instead of throwing and catching the ball), and 15 medicine ball rotations. WODs were often intentionally self-limiting (e.g., AMRAP in 20 min) so that individuals with higher levels of fitness could complete a greater volume of work but all participants started and ended at the same time. We rarely used WODs that involved RFT as this drew attention to participants with more significant impairments or lower fitness levels.

### Educational and motivational components of the program

Before the first exercise session, the lead coach provided education on safety with exercise, the community aspect of CrossFit, and how to use the Rate of Perceived Exertion scale to rate exercise intensity. Participants were oriented to the facility and learned how to safely use aerobic exercise equipment. Prior to each session, coaches described and demonstrated all exercises, including adapted versions for those with different abilities, including individuals with paraplegia, tetraplegia and those who were ambulatory. Select sessions ended with non-exercise components including mindfulness (sessions 13 and 20) and general nutritional advice from a registered dietician (session 11).

Participants were provided monthly newsletters (6 total), which provided education on CrossFit and its adaptive divisions, safety with exercise, local opportunities to engage in exercise, SCI-specific exercise guidelines [18], how to make a workout (with examples), and hydration with exercise. The newsletters also provided information about the coaches and select participants (e.g., where they were from, hobbies, etc.). While additional exercise was encouraged, no specific exercise was prescribed by coaches outside of sessions.

Various behavioral change techniques were used to enhance motivation and enjoyment. Self-efficacy [19] was targeted through vicarious experiences (observing coaches and other participants perform exercise), mastery experiences (the exercise program started with simple exercises at a low volume to allow early participant success), and verbal persuasion (coaches and fellow participants provided verbal encouragement and “fist bumps” during and after sessions) [20]. Social support was targeted through education on exercise performance, encouragement from coaches and fellow participants, providing all necessary equipment to safely participate in exercise, and the involvement of CPs, team and partner WODs [21]. Additionally, each session incorporated music chosen by participants.

### Demographic & feasibility measures

Demographic data was collected prior to starting the exercise program. Measures of feasibility included recruitment, retention, attendance, safety, and fidelity (exercise intensity). Recruitment rate was the number of participants who underwent T1 and/or T2 assessments and participated in at least one exercise session compared to the number of prospective participants contacted. Retention rate was the number of participants who completed T2 and T3 assessments compared to the number of participants who completed T1. Attendance rate was the number of total exercise sessions attended divided by the number of available exercise sessions for each participant (i.e., 49 if a participant began the study at T1). To mimic real-word community programs, there were no attendance requirements. Rather, participants were encouraged to attend as many sessions as possible. Safety was measured as the number of adverse events that occurred as a result of the program and impacted exercise participation.

Fidelity was measured through session Rating of Perceived Exertion (session-RPE) [22]. Session-RPE was pragmatically chosen because large group data can be captured efficiently and donning/doffing heart rate monitors would increase the pre-exercise burden for both participants and coaches. RPE has been found independent of exercise mode and the level of SCI [23]. At the end of each session participants were shown a 0 to 10 RPE scale, then asked to verbally answer “how was your workout?” using the scale. Prior to beginning the study we operationally defined high-intensity as 5 (“hard”) or greater, which has been shown to correspond to anaerobic thresholds for people with and without SCI [23]. Adverse events and Session-RPE were the only participant data captured during exercise sessions.

### Physical & psychosocial outcome measures

At each assessment point, physical and psychosocial data were collected. Cardiovascular endurance was measured via the 6-Min Arm Test (pSCI) [24] or 6-Min Walk Test (CPs only) [25]. Muscular strength was measured via hand-held dynamometry of the upper extremities [26] (pSCI) and/or Five Times Sit To Stand Test [27]. Walking speed was measured via the 10-Meter Walk Test [28] for both self-selected and fast speeds. Average power over one minute and anaerobic peak power (measured in watts) was measured with a ski ergometer (SkiErg®). Participants were instructed to “pull as hard and as fast as you can on each pull over the course of one minute”. Test position (i.e., standing or seated in chair/wheelchair) and damper setting were standardized across assessments.

Self-efficacy was measured via Exercise Self-Efficacy Scale (pSCI) [29] and Self-Efficacy for Exercise Scale (CPs) [30]. Social support was measured via Social Support and Exercise Survey [31]. Perceived physical function was measured via short forms of the Spinal Cord Injury-Functional Index (pSCI) [32]. Health-related quality of life was measured via short forms of the Spinal Cord Injury—Quality of Life (pSCI) [33] or Rand 36-item Short-Form 36 (SF-36) (CPs) [34].

A global rating of change (GRC) scale had participants rate their perceived level of change in weekly physical activity levels, ability to walk, push, or move fast, and ability to walk, push, or move for a long period of time compared to when they started the exercise program.

All assessments were conducted by the same trained physical therapists involved in the exercise program (RH or KH).

### Data analysis

Descriptive statistics were used to present demographic and feasibility data. For physical and psychosocial outcomes, pSCI and CP data were analyzed separately. The Wilcoxon signed-rank test was used to examine within-group (pSCI or CPs) differences for those who completed T1, T2, and T3 assessments. Effect size, *r*, was calculated [35, 36]. Effect sizes of 0.5, 0.3, and 0.1 were considered large, medium, and small, respectively [37]. Alpha was set at ≤0.05. IBM SPSS Statistics for Macintosh, Version 26.0 (IBM SPSS, Chicago, IL) was used for data analysis.

## Results

The study took place between February and November 2022. The program contained 108 different exercises (Supplementary Appendix 1). The most commonly performed were aerobic (row, ski, bike, propulsion, walking; 61.2% of sessions), burpees (32.7%), rope swings (28.6%), medicine ball rotations (26.5%), medicine ball wall ball (24.5%), u-turns (20.4%), medicine ball slams (20.4%) medicine ball ground to shoulder (18.4%), hoists (16.3%), seal jacks (16.3%), battle ropes (14.3%), forward sled drags (14.3%), free weight side bends (14.3%), barbell bench press (12.2%) and thoracic mobility (12.2%). In regard to workout structure, intervals were programmed most frequently (44.9% of sessions) followed by AMRAPs (34.7%), partner WODs (32.7%), EMOMs (26.5%), aerobic (20.4%), mobility (20.4%), strength (10.2%) and team WODs (10.2%).

### Demographics & Feasibility

Demographic data of participants are displayed in Table 1. Participants with SCI were predominantly older white males who used manual or power wheelchairs for mobility. CPs were mostly female spouses of pSCI. Participants lived in socioeconomically disadvantaged areas of the U.S. as noted by ADI’s ≥72 (72nd percentile in terms of disadvantage).

**Table 1:** Participant demographics.

	Participants		
Descriptor	Total(*n* = 20)	pSCI (*n* = 14)	CPs (*n* = 6)
Age In Years, Median (IQR)	60 (15.8)	59.5 (15.3)	61.5 (24.5)
Years Since SCI, Median (IQR)	–	10.1 (21.8)	–
Miles From Facility, Median (IQR)	14.4 (16.3)	14.4 (14.8)	15.1 (28.2)
ADI National Percentile, Median (IQR)^a^	75 (27)	77 (25)	72 (42)
	n (%)	n (%)	n (%)
Sex			
Female	6 (30%)	2 (14%)	4 (67%)
Male	14 (70%)	12 (86%)	2 (33%)
Race/Ethnicity			
American Indian	–	–	–
Asian	1 (5%)	1 (7%)	–
Black or African American	1 (5%)	1 (7%)	–
Hispanic or Latino	–	–	–
White	18 (90%)	12 (86%)	6 (100%)
Household Income			
<$25,000	6 (30%)	4 (29%)	2 (33%)
$25,000–$49,999	2 (10%)	2 (14%)	–
$50,000–$99,999	9 (45%)	6 (43%)	3 (50%)
$100,000–$149,999	3 (15%)	2 (14%)	1 (17%)
Marital Status			
Married	13 (65%)	8 (57%)	5 (83%)
Not Married	7 (35%)	6 (43%)	1 (17%)
Primary Means of Mobility			
Manual Wheelchair	6 (30%)	6 (43%)	–
Power Wheelchair	4 (20%)	4 (29%)	–
Walking with no assistive device	6 (30%)	–	6 (100%)
Walking with assistive device	4 (20%)	4 (29%)	–
Medical History			
Arthritis	2 (10%)	1 (7%)	1 (17%)
Cancer	5 (25%)	3 (21%)	2 (33%)
Diabetes	6 (30%)	4 (29%)	2 (33%)
Heart disease	2 (10%)	–	2 (33%)
Hypertension	7 (35%)	6 (43%)	1 (17%)
Neuropathy	2 (10%)	2 (14%)	–
Severity of Spinal Cord Injury			
C5-8 ASIA A	–	2 (14%)	–
C5-8 ASIA B	–	2 (14%)	–
C5-8 ASIA C	–	3 (21%)	–
T1-S5 ASIA A	–	3 (21%)	–
T1-S5 ASIA C	–	2 (14%)	–
T5 ASIA D	–	1 (7%)	–
L3 ASIA D	–	1 (7%)	–
Relationship to pSCI			
Spouse	–	–	4 (67%)
Partner	–	–	1 (17%)
Daughter	–	–	1 (17%)
Assistance Provided to pSCI			
ADLs	–	–	4 (67%)
IADLs	–	–	6 (100%)
Physical Activity Compared to Peers^b^			
Much More Active	1 (5%)	1 (7%)	–
More Active	8 (40%)	5 (36%)	3 (50%)
About as Active	5 (25%)	3 (21%)	2 (33%)
Less Active	4 (20%)	3 (21%)	1 (17%)
Much Less Active	2 (10%)	2 (14%)	–

*pSCI* person/people with spinal cord injury, *CP* CP(s), *SCI* spinal cord injury, *ADI* Area Deprivation Index, *ASIA* American Spinal Injury Association (ASIA) International Standards for Neurological Classification of Spinal Cord Injury, *ADLs* activities of daily living (e.g., bathing, feeding, self-care), *IADLs* instrumental activities of daily living (e.g. driving, meal preparation, housekeeping).

^a^Area Deprivation Index with national percentiles [47, 48] provides a metric of socioeconomic disadvantage compared on a national level.

^b^A single item was used to assess perceived physical activity levels: “Compared to other people your own age, do you think you are…much more active, more active, about as active, less active, or much less active? [49]”.

**Fig. 1:**
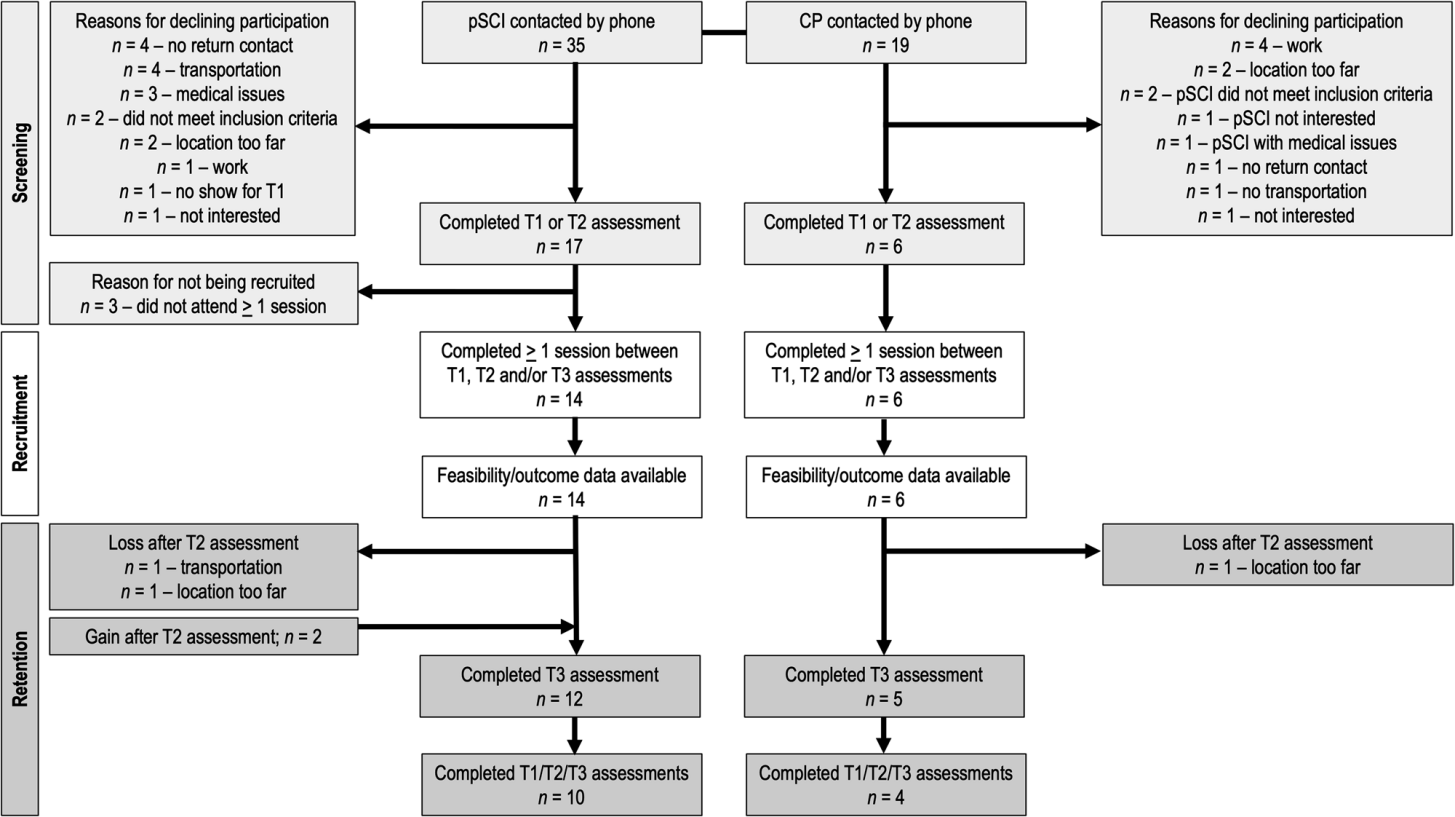
Flow diagram of participant screening, recruitment and retention. pSCI person/people with spinal cord injury, CP CP(s), T1 assessment occurring before the program, T2 assessment occurring after 13 weeks of the program, T3 assessment occurring after 25 weeks of the program.

A flow diagram with screening, recruitment, and retention is displayed in Fig. 1. Recruitment rates were 40% (14/35) for pSCI and 32% (6/19) for CPs. Healthcare providers provided contact information for 27 of the 35 pSCI contacted in the present study. Contact information was also received through word of mouth from other participants [6] and a news article on the program [2]. Retention rates were 83% (10/12) for pSCI and 80% (4/5) for CPs.

**Fig. 2:**
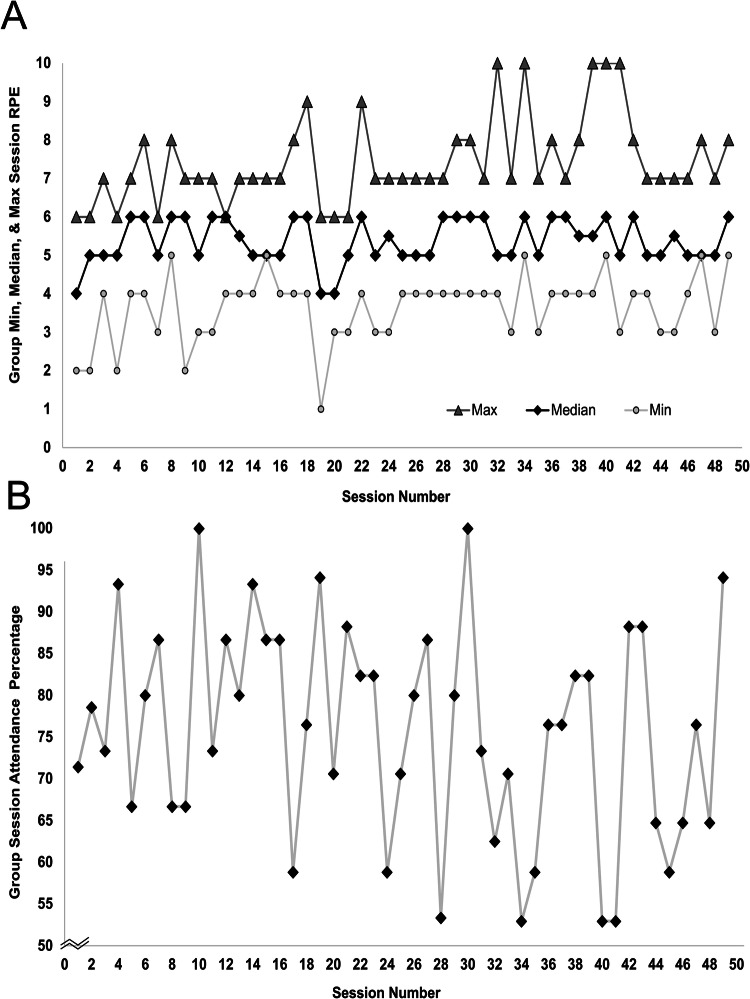
Rating of perceived exertion and participant attendance across 49 exercise sessions. For (**A**), maximum ratings of perceived exertion are indicated by triangles, medians by diamonds, and minimums by circles. **B** displays average group attendance across 49 sessions.

Exercise session attendance rates and group session-RPE values are displayed in Fig. 2. Average (SD) program attendance was 73% (22%) for all participants, 77% (19%) for pSCI and 61% (24%) for CPs. Median (25 and 75% percentiles) session-RPE was 5 (5, 6) for all participants, 6 (5, 6) for pSCI and 5 (4.75, 5) for CPs, indicating exercise met our predefined criteria for high-intensity. One adverse event occurred during the program: self-identified autonomic dysreflexia requiring 10 min of inactivity prior to returning to exercise.

**Fig. 3:**
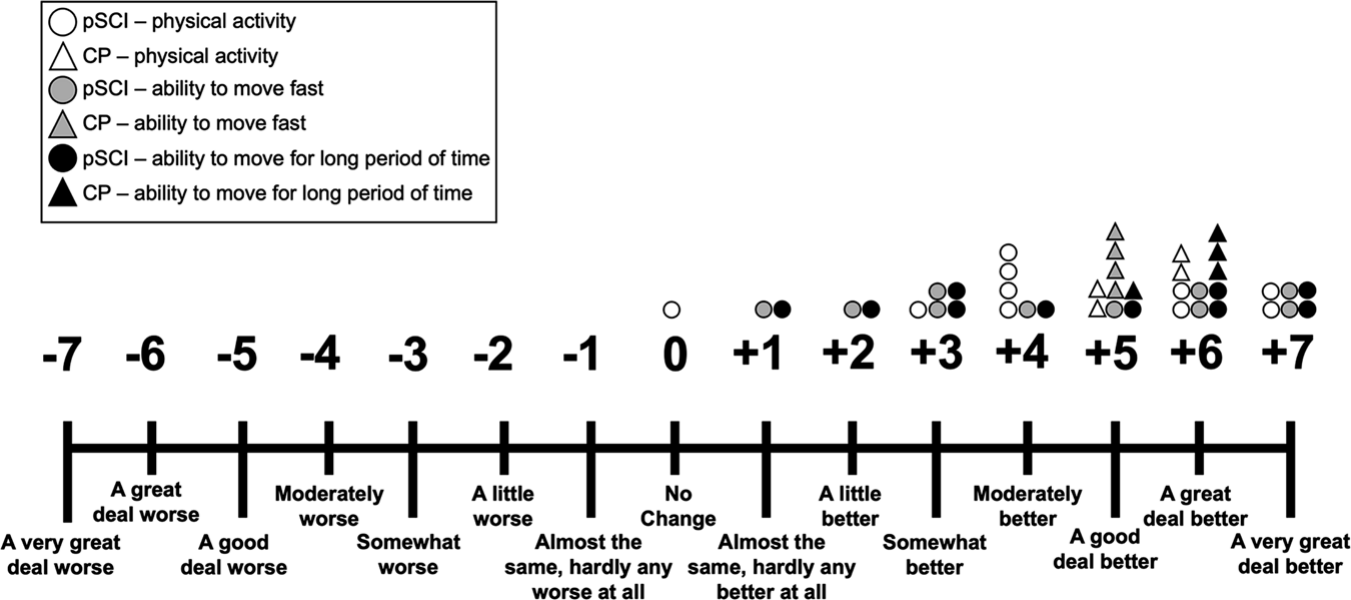
GlObal rating of change. pSCI person/people with spinal cord injury, CP CP(s), After the 25 week program, participants were asked to answer the following using a 15-point Global Rating of Change Scale: “Compared to when you started the exercise program, has there been any change in amount of *physical activity* you perform in an average week, has there been any change in your ability to walk, push, or *move fast*, and has there been any change in your ability to walk, push, or *move for a long period of time*?” Each shape (i.e., circle, triangle) represents a single participant.

For the ten pSCI who completed T1, T2, and T3 assessments, the median ratings on the GRC scale were +5.5 (3.75, 6.25) for perceived change in weekly physical activity, +4.5 (2.75, 6.25) for ability to move fast and +4.5 (2.75, 6.25) for ability to move for a long period of time, respectively (Fig. 3). The four CPs who completed T1, T2, and T3 assessments rated their changes as +5.5 (5, 6), +5 (5, 5), and +6 (5.25, 6), for weekly physical activity, ability to move fast, and ability to move for a long period of time, respectively (Fig. 3).

### Physical & psychosocial outcomes

Tables 2, 3 display physical and psychosocial outcomes for pSCI and CPs, respectively. These tables include the median of differences between assessments and within-group differences with effect sizes between T1 and T3 assessments. For pSCI, large effect sizes were observed for cardiovascular endurance, anaerobic power, lower extremity strength, and social support for exercise from friends. As a group, CPs observed large, non-statistically significant improvements in cardiovascular endurance, peak power, fast walking speed, lower extremity strength, exercise self-efficacy, social support for exercise from family and friends, and all components of quality life from the SF-36.

**Table 2:** Physical & psychosocial outcomes for people with spinal cord injury^a^.

			*n*	T1	T2	Δ	*n*	T2	T3	Δ	*n*	T1	T3	Δ	Effect size (*r*)^b^
Physical	Endurance	6MAT (6 to 20 RPE rated after sixth minute of test)	12	13.5 (9)	12 (8.8)	−1	12	10.5 (4.3)	11 (3)	.5	10	13.5 (9)	12 (6)	−2.5	0.55*
	Power^c^	Anaerobic Average Power (W)	12	49 (59)	67 (98)	17	12	67 (70)	85 (59)	4	10	49 (74)	73 (87)	20.5	0.63*
		Anaerobic Peak Power (W)	12	62 (81)	88 (131)	17	12	88 (87)	107 (83)	7	10	62 (107)	94 (133)	28.5	0.56*
	Speed	Self-Selected Walking Speed (m/s)	4	0.49 (0.2)	0.66 (.6)	0.10	4	0.66 (0.61)	0.63 (.52)	−0.04	3	0.53 (.23)	0.84 (.55)	0.16	0.3
		Fast Walking Speed (m/s)	4	0.64 (0.19)	0.83 (.63)	0.17	4	0.83 (.51)	0.87 (.55)	−0.03	3	.71 (.21)	0.92 (0.7)	0.16	0.3
	Strength	5xSTS (s)	2	14.2	9.4	−4.78	2	9.4	7.6	−1.8	2	14.2	7.6	−6.6	**0.95**
		R Sh Flexors (lbs)	12	12.7 (6.2)	15 (5.6)	0.4	12	14.5 (5.6)	12.7 (7)	0.3	10	12.7 (5.6)	12.7 (6.4)	0.7	0.31
		L Sh Flexors (lbs)	12	13.2 (5.6)	13.6 (6.6)	1	12	13.6 (5.8)	14.5 (5.1)	1.3	10	12.2 (5.8)	14.5 (6.2)	2.8	0.49
		R Sh Extensors (lbs)	12	10.4 (3.9)	14.1 (5.1)	1.3	12	14.1 (4.9)	10 (3.7)	−1	10	10.4 (3.5)	10.9 (4.5)	1.1	0.24
		L Sh Extensors (lbs)	12	9.5 (3.5)	10.9 (4.5)	2.1	12	10.9 (2.9)	11.3 (2.1)	0.2	10	9.5 (3.7)	11.8 (3.3)	2	0.42
		R El Flexors (lbs)	12	19.1 (11.3)	20 (8)	1.6	12	21.8 (7.8)	20.9 (6.6)	1	10	18.1 (12.1)	20.9 (6.6)	2.7	0.26
		L El Flexors (lbs)	12	19.5 (9.7)	23.1 (7)	1.5	12	25.4 (7)	22.7 (7)	−1.2	10	19.5 (11.1)	21.8 (9.3)	3.7	0.42
		R El Extensors (lbs)	12	10.4 (9.1)	12.2 (8.4)	1.5	12	15.4 (7)	13.6 (7.2)	−0.3	10	9.5 (9.3)	11.8 (8.2)	2	0.22
		L El Extensors (lbs)	12	10.9 (8.4)	11.3 (7.4)	0.4	12	14.1 (7.8)	13.6 (6)	0	10	10 (9.3)	11.3 (7)	0	0.12
Psychosocial		Exercise Self-Efficacy^d^	12	35.5 (7.3)	36 (6)	0	12	37 (6.8)	34.5 (5.8)	−1	10	35.5 (8.8)	34 (6)	0	0.06
		Social Support for Exercise – Family^e^	12	22 (13.3)	27.5 (12)	4	12	27.5 (12)	21.5 (15)	−6	10	22 (10.5)	23 (20)	−2	0.08
		Social Support for Exercise – Friends^e^	12	13.5 (7.5)	30.5 (14.3)	14.5	12	28 (17.8)	20 (16)	−3.5	10	15 (9)	20 (14)	7	0.63*
	SC- FI^f^	Self-care	12	55.5 (8.7)	55 (8.9)	0	12	56.1 (6.5)	56.9 (10.7)	0	10	55.5 (10.5)	56.4 (8.8)	0.9	0.28
		Basic mobility	12	55.5 (9)	55.5 (12)	0.6	12	57.6 (8.4)	59.4 (8.2)	−0.2	10	55.5 (7.5)	56.4 (9.9)	1.8	0.25
		Manual w/c	4	59.3 (10)	60.2 (8.1)	−2	6	60.2 (7.2)	60.7 (6.4)	−0.4	4	59.3 (9.9)	59 (7.3)	−3.4	0.39
		Power w/c	4	47.7 (9.4)	50 (15.3)	2.5	3	46.1 (18)	45.3 (12.5)	−0.8	4	49.8 (10.4)	50.2 (11.8)	1.2	0.38
		Ambulation	4	63.7 (5)	64 (5)	0.2	4	67 (10.1)	66.3 (6.3)	−0.7	3	64.2 (6.3)	64.9 (4.3)	0.7	0
	SCI-QOL^g^	Positive Affect	12	53.3 (9.3)	57 (11.5)	1.1	12	57 (11.3)	57 (11.9)	−0.8	10	53.7 (9.9)	55 (13.5)	−0.4	0.16
		Resilience	12	52.1 (14)	50.8 (17.8)	0	12	53.6 (19.6)	49.4 (17.8)	0	10	52.1 (15.7)	49.4 (20.9)	0	0.02
		Self-esteem	12	45.7 (14.2)	48.2 (11.3)	0.5	12	51.1 (13.2)	47.3 (21.3)	0	10	47.2 (17.6)	44.3 (17.9)	0	0.09
		Independence	12	52.4 (17)	57 (20.2)	1.5	12	57 (14.5)	55.6 (17.3)	1	10	53.9 (17)	53.1 (17.8)	1.4	0.23
		Stigma	12	55.9 (4.3)	54.1 (8.5)	−0.3	12	54.1 (6.9)	55.9 (9.1)	0.8	10	55 (6.5)	55.9 (7.7)	0	0.11

*T1* assessment occurring before the program, *T2* assessment occurring after 13 weeks of the program, *T3* assessment occurring after 25 weeks of the program, Δ median of differences, 6MAT 6-Min Arm Test, *W* watts, *5xSTS* Five Times Sit to Stand, *m/s* meters per second, *kg*, kilogramsf, *R* right, *L* left, *Sh* shoulder, *El* elbow, *5xSTS* Five Times Sit to Stand, *s* seconds, *SCI-FI* Spinal Cord Injury Functional Index, *w/c* wheelchair, *SCI-QOL* spinal cord injury-quality of life.

^a^Data presented as median (IQR).

^b^Wilcoxon signed-rank test used to assess within group differences between T1 and T3. Effect size calculated using resulting test statistic, Z, from Wilcoxon test. Boldface indicates large effect size (r = ≥0.5).

^c^Measured via maximal one minute effort on SkiErg®

^d^Measured via Exercise Self-Efficacy Scale. Scores range from 10 to 40 with higher scores indicating greater self-efficacy for exercise.

^e^Measured via Sallis Social Support and Exercise Survey. There are two scores, one for family and one for friends that range from 10 to 50 with higher scores indicating greater social support for exercise.

^f^SCI-FI is scored using a standardized T metric with a mean of 50 and a standard deviation of 10. Higher scores indicate better function. A T-score of 60 for Manual Wheelchair Mobility indicates an individual is functioning at one standard deviation above the mean of pSCI [33].

^g^SCI-QOL is scored using a standardized T metric with a mean of 50 and a standard deviation of 10. Higher scores indicate a greater amount of the construct being measured. A T-score of 40 for Positive Affect indicates an individual is functioning at one standard deviation below the mean of pSCI [33].

*Statistical significance at *p* ≤ 0.05.

**Table 3:** Physical & psychosocial outcomes for cps of people with spinal cord injury^a^.

			*n*	T1	T2	Δ	*n*	T2	T3	Δ	*n*	T1	T3	Δ	Effect size (*r*)^b^
Physical	Endurance	6MWT (m)	5	475 (63.1)	538.3 (77.7)	56.7	5	538.3 (75.9)	556.1 (85)	16	4	477.9 (84.8)	561.4 (104.9)	65.1	**0.65**
	Power^c^	Anaerobic Average Power (W)	5	94 (155)	152 (132)	10	5	152 (140)	168 (153)	2	4	140 (155)	183 (124)	28	0.38
		Anaerobic Peak Power (W)	5	124 (224)	216 (198)	32	5	216 (211)	237 (212)	5	4	187 (236)	252 (170)	33	**0.52**
	Speed	Self-Selected Walking Speed (m/s)	5	1.24 (0.29)	1.23 (0.41)	−0.04	5	1.18 (.37)	1.22 (.26)	0.08	4	1.23 (.25)	1.2 (.34)	0	0.13
		Fast Walking Speed (m/s)	5	1.89 (0.39)	1.84 (0.28)	0.03	5	1.84 (.29)	1.91 (.46)	−0.08	4	1.9 (.44)	1.96 (.42)	.05	**0.65**
	Strength	5xSTS (s)	5	11.4 (5.3)	9.2 (4.7)	−2.6	5	8.2 (4.2)	9.56 (3.4)	0	4	11.9 (7.4)	9.7 (4)	−2	**0.65**
Psychosocial		Exercise Self-Efficacy^d^	5	41 (30)	49 (28.5)	13	5	60 (26.5)	67 (14.5)	−2	4	42.5 (37.5)	68.5 (7.5)	26.5	**0.52**
		Social Support for Exercise – Family^e^	5	15 (6.5)	42 (21.5)	27	5	42 (28)	35 (17.5)	4	4	15 (6.8)	40 (13)	24	**0.65**
		Social Support for Exercise – Friends^e^	5	10 (0.5)	36 (30.5)	25	5	36 (32)	15 (3)	−24	4	10 (.8)	14.5 (3.3)	4.5	**0.65**
	SF-36^f^	Physical Function	5	50 (52.5)	75 (30)	10	5	75 (30)	80 (37.5)	5	4	50 (48.8)	82.5 (38.8)	20	**0.57**
		Role Limitations Due to Physical Health	5	25 (62.5)	75 (37.5)	50	5	100 (37.5)	100 (37.5)	0	4	12.5 (81.3)	87.5 (43.8)	50	**0.58**
		Role Limitations Due to Emotional Problems	5	0 (83.3)	66.7 (100)	0	5	100 (100)	100 (50)	0	4	0 (75)	100 (75)	50	**0.5**
		Energy/Fatigue	5	20 (42.5)	60 (30)	30	5	55 (50)	60 (35)	5	4	17.5 (27.5)	62.5 (27.5)	40	**0.65**
		Emotional Well-being	5	48 (32)	64 (18)	16	5	72 (20)	76 (26)	8	4	40 (28)	76 (18)	26	**0.5**
		Social Functioning	5	62.5 (25)	62.5 (56.3)	12.5	5	87.5 (56.3)	75 (37.5)	0	4	56.3 (31.3)	81.3 (37.5)	37.5	**0.58**
		Pain	5	45 (56.3)	67.5 (38.8)	12.5	5	57.5 (38.8)	67.5 (45)	0	4	38.8 (61.3)	72.5 (50)	22.5	**0.57**
		General Health	5	55 (30)	60 (25)	10	5	60 (22.5)	55 (32.5)	−5	4	52.5 (38.8)	60 (36.3)	5	**0.58**

*T1* assessment occurring before the program, *T2* assessment occurring after 13 weeks of the program, *T3* assessment occurring after 25 weeks of the program, *Δ* median of differences, *6MWT* Six Minute Walk Test, *m* meters, *W* watts, *m/s* meters per second, *5xSTS* Five Times Sit to Stand, *s* seconds, *SF-36* 36-Item Short Form Survey.

^a^Data presented as median (IQR).

^b^ Wilcoxon signed-rank test used to assess within group differences between T1 and T3. Effect size calculated using resulting test statistic, Z, from Wilcoxon test. Boldface indicates large effect size (*r* = ≥ 0.5).

^c^Measured via maximal one minute effort on SkiErg®.

^d^Measured via Self-Efficacy for Exercise scale. Scores range from 0–90 with higher scores indicating greater self-efficacy for exercise.

^e^Measured via Sallis Social Support and Exercise Survey. There are two scores, one for family and one for friends that range from 10 to 50 with higher scores indicating greater social support for exercise.

^f^Each domain in the SF-36 is scored 0 to 100 with higher scores indicating a greater state of health.

## Discussion

To date, this is the first study examining HIFT in pSCI or CPs. Results indicate that 49 sessions of HIFT over 25 weeks can be safely implemented for CPs and pSCI, including those with paraplegia, tetraplegia, and those who are ambulatory. Despite reported barriers to exercise amongst pSCI, attendance levels in the present study were high and comparable to previous work utilizing twice weekly multimodal exercise performed at a moderate to high-intensity [2]. Whether attendance levels would sustain beyond 25 weeks is not known. Results from a nine month intervention found similar attendance rates, but a lower retention rate (52% [2] compared to the present study’s 83% retention rate for pSCI). Methods employed in the present study, including varied workouts, over one hundred exercises, partner and team-based workouts, educational and motivational components and opportunities to exercise alongside CPs may have contributed to the high attendance and retention rates observed for pSCI. These findings are also supported by the perceived benefits of the program as observed with the GRC scale.

While attendance was high, recruitment of participants was a challenge. A review of recruitment rates for people with neurologic disability found that only 42% of those screened enroll in exercise studies, with commonly cited reasons including feelings of exclusion and lack of transportation [38]. Though over half of pSCI contacted for this study had CPs, less than one-third were successfully recruited. The primary reasons for declining participation amongst pSCI were transportation and medical issues, whereas for CPs, work was most common. Offering exercise programs at the start or end of the typical workday may facilitate greater participation, particularly amongst pSCI and/or CPs who work during the day. Alternatively, a partner-based home-based exercise program may be better suited for both parties as this would remove the common SCI transportation barrier and allow exercise to occur outside of work hours.

To our knowledge, this study is the first to use session-RPE to gauge exercise intensity in a group of pSCI. The median RPE met our criteria of ≥5 out of 10, but in a given session, RPE values ranged from 1 to 10 (Fig. 2). This means exercise intensity was too low or perhaps too high at times for select participants. While moderate and even light intensity exercise is beneficial, HIIT has been found to be just as effective as moderate intensity exercise for improving cardiovascular endurance and muscular strength but with the added benefit of taking less than half the training time [39]. In pSCI HIIT has also been found to be more enjoyable than moderate intensity continuous exercise [40]. While there is a trend for exercise interventions to prioritize higher intensities, these do briefly increase the risk cardiovascular events [41] and musculoskeletal injuries [42], which is particularly important for pSCI who are at heightened risk for cardiovascular disease and may utilize their upper extremities for transfers and mobility. While the present study only had one adverse event that interfered with exercise (i.e., transient autonomic dysreflexia), the safety of HIFT for pSCI requires further investigation. Obtaining medical clearance, having trained exercise coaches, and providing participant education regarding safe exercise is likely paramount to safely implementing group based HIFT for pSCI. Future studies that pair session-RPE with other metrics of intensity (e.g., heart rate, power output, load) would provide valuable data on the safety, validity, and efficacy of using session-RPE to prescribe exercise in pSCI.

While not powered to detect statistically significant differences, pSCI demonstrated significant improvements in several physical outcomes including cardiovascular endurance, and average anaerobic and peak power output on the SkiErg®. The SkiErg® was the most commonly programmed aerobic equipment (Supplementary Appendix 2). When given the choice, the majority of participants who utilized wheelchairs, including those with paraplegia and tetraplegia, chose skiing as their form of aerobic activity, rather than arms-only rowing or propulsion. This aligns with previous findings that pSCI find arm-only exercise equipment to be safer than equipment that also provides passive movement of the legs [43]. The ski ergometer’s attributes were appealing to both participants and coaches as the equipment is inclusive (all pSCI and CPs could utilize), uses opposing muscle groups to those used in wheelchair propulsion, and captures valuable data (monitor provides immediate feedback on, and records, speed, distance, and power output). The present study suggests the SkiErg® may be used to capture changes in anaerobic power output for pSCI and CPs who walk or utilize a wheelchair. However, caution must be used as metrics of reliability and validity have not been established.

Both pSCI and CPs reported improvements in social support for exercise. Participants with SCI had a significant and large improvement in their perceived social support from friends, whereas CPs reported increased support from both family and friends. Social support for exercise is a strong predictor of exercise adherence [44]. In adults without disability, CrossFit was found to foster a greater sense of community than traditional group exercise classes [45, 46] and individual exercise [45], though it was not predictive of gym attendance [46]. Participants in the present study may have felt supported by others in the program who shared similar life experiences. This sense of belonging may have facilitated regular attendance. Interestingly, pSCI did not report improvements in support from family, despite several having CPs actively participate in the program. In contrast, CPs felt more supported by family, perhaps because they were involved in the program rather than the typical facilitator role in rehabilitation settings. Future exercise studies should consider involving CPs to better understand their role in promoting health in themselves and pSCI.

### Limitations

The present study had a small, predominantly white male sample of pSCI, a lack of a control group, and assessments performed by unmasked assessors. These limit generalizability of study findings to other pSCI, do not allow causation to be inferred and introduce potential bias into outcome assessments. The lack of a control or comparison group means we cannot attribute differences in outcomes to the intervention alone, as other factors, both measured and unmeasured, may have impacted outcomes. An additional limitation is the lack of individual data regarding performance during exercise sessions (e.g., loads used or repetitions performed). This was an unfortunate tradeoff when supervising large group exercise sessions but must be considered when interpreting study findings. The standard of recovery, American Spinal Injury Association (ASIA) International Standards for Neurological Classification of Spinal Cord Injury was not assessed post-program and thus if any changes in spinal cord recovery occurred, these would have been missed. In this study, physical measures were chosen based on traditional rehabilitation outcomes and may not have been optimal for a HIFT program. Future research should investigate the psychometrics of inclusive HIFT-specific work capacity tests that utilize equipment in commercial (e.g., CrossFit) rather than rehabilitation facilities. Establishing reliability and validity in these tests will be necessary as research on HIFT for pSCI becomes more prevalent. Finally, this study did not examine HIFT’s impact on cardiometabolic outcomes (e.g., lipid profiles, insulin sensitivity, blood pressure) which play an integral role in the health of pSCI.

## Conclusion

The present study found that 49 sessions of HIFT across 25-weeks is feasible for pSCI and CPs, and potentially leads to improvements in physical and psychosocial health for both groups. HIFT, especially CrossFit, is widely available and may be a viable exercise option for pSCI in other communities. Subsequent studies should examine HIFT and other forms of exercise such as HIIT and compare factors such as attendance rates, health outcomes, cost-effectiveness and sustainability.

### Supplementary information

Supplementary Material Legends

Appendix 1

Appendix 2

Dataset

## Data Availability

Data from this study are available from the corresponding author on reasonable request.

## References

[1] Piercy KL, Troiano RP, Ballard RM, Carlson SA, Fulton JE, Galuska DA (2018). The physical activity guidelines for Americans. JAMA.

[2] Hicks AL, Martin KA, Ditor DS, Latimer AE, Craven C, Bugaresti J (2003). Long-term exercise training in persons with spinal cord injury: effects on strength, arm ergometry performance and psychological well-being. Spinal Cord.

[3] Nightingale TE, Rouse PC, Walhin JP, Thompson D, Bilzon JLJ (2018). Home-based exercise enhances health-related quality of life in persons with spinal cord injury: a randomized controlled trial. Arch Phys Med Rehabil.

[4] van der Scheer JW, Ginis KAM, Ditor DS, Goosey-Tolfrey VL, Hicks AL, West CR (2017). Effects of exercise on fitness and health of adults with spinal cord injury: a systematic review. Neurology.

[5] Miller LE, Herbert W (2016). Health and economic benefits of physical activity for patients with spinal cord injury. Clin Outcomes Res [Internet].

[6] Conti A, Clari M, Nolan M, Wallace E, Tommasini M, Mozzone S (2019). The relationship between psychological and physical secondary conditions and family caregiver burden in spinal cord injury: a correlational study. Top Spinal Cord Inj Rehabil [Internet].

[7] Tough H, Brinkhof MWG, Fekete C (2020). Is informal caregiving at odds with optimal health behaviour? A cross-sectional analysis in the caregiving partners of persons with spinal cord injury. Heal Psychol Behav Med.

[8] Schulz R, Czaja SJ, Lustig A, Zdaniuk B, Martire LM, Perdomo D (2009). Improving the quality of life of caregivers of persons with spinal cord injury: A randomized controlled trial. Rehabil Psychol.

[9] Lavela SL, Landers K, Etingen B, Karalius VP, Miskevics S. Factors related to caregiving for individuals with spinal cord injury compared to caregiving for individuals with other neurologic conditions. J Spinal Cord Med. 2015;38:505–14. 10.1179/2045772314Y.0000000240 PMC4612206 24993244

[10] Sallis JF, Cervero RB, Ascher W, Henderson KA, Kraft MK, Kerr J (2006). An ecological approach to creating active living communities. Annu Rev Public Heal.

[11] Sallis JF, Floyd MF, Rodríguez DA, Saelens BE (2012). Role of built environments in physical activity, obesity, and cardiovascular disease. Circulation.

[12] Feito Y, Heinrich KM, Butcher SJ, Poston WSC (2018). High-intensity functional training (HIFT): Definition and research implications for improved fitness. Sports.

[13] Buckley S, Knapp K, Lackie A, Lewry C, Horvey K, Benko C (2015). Multimodal high-intensity interval training increases muscle function and metabolic performance in females. Appl Physiol Nutr Metab.

[14] Kercher VMM, Kercher K, Levy P, Bennion T, Alexander C, Amaral PC (2023). 2023 fitness trends from around the globe. ACSMs Health Fit J.

[15] Heinrich KM, Patel PM, O’Neal JL, Heinrich BS (2014). High-intensity compared to moderate-intensity training for exercise initiation, enjoyment, adherence, and intentions: an intervention study. BMC Public Health.

[16] Hendersson S. Morning Chalk Up. 2018 [cited 2024 Feb 8]. Crossfit’s explosive affiliate growth by the numbers. Available from: https://morningchalkup.com/2018/10/23/crossfits-explosive-affilaite-growth-by-the-numbers/.

[17] MacMillan S. CrossFit gym offers program for people with spinal-cord injuries [Internet]. 2018 [cited 2024 Feb 8]. Available from: https://www.cbc.ca/news/canada/prince-edward-island/pei-spinal-cord-injury-crossfit-1.4637999#:~:text=.

[18] Ginis KAM, Van Der Scheer JW, Latimer-Cheung AE, Barrow A, Bourne C, Carruthers P (2018). Evidence-based scientific exercise guidelines for adults with spinal cord injury: an update and a new guideline. Spinal Cord.

[19] Bandura A. Social foundations of thought and action. Englewood Cliffs, NJ. 1986;1986.

[20] Liguori G (2020). ACSM’s guidelines for exercise testing and prescription.

[21] Wills TA, Shinar O. Measuring perceived and received social support. 2000;

[22] Foster C, Florhaug JA, Franklin J, Gottschall L, Hrovatin LA, Parker S (2001). A new approach to monitoring exercise training. J Strength Cond Res.

[23] Hutchinson MJ, Kouwijzer I, de Groot S, Goosey-Tolfrey VL (2021). Comparison of two Borg exertion scales for monitoring exercise intensity in able-bodied participants, and those with paraplegia and tetraplegia. Spinal Cord.

[24] Hol AT, Eng JJ, Miller WC, Sproule S, Krassioukov AV (2007). Reliability and validity of the six-minute arm test for the evaluation of cardiovascular fitness in people with spinal cord injury. Arch Phys Med Rehabil.

[25] Rikli RE, Jones CJ (1998). The reliability and validity of a 6-minute walk test as a measure of physical endurance in older adults. J Aging Phys Act.

[26] Sisto SA, Dyson-Hudson T (2007). Dynamometry testing in spinal cord injury. J Rehabil Res Dev.

[27] Khuna L, Thaweewannakij T, Wattanapan P, Amatachaya P, Amatachaya S (2020). Five times sit-to-stand test for ambulatory individuals with spinal cord injury: a psychometric study on the effects of arm placements. Spinal Cord.

[28] Scivoletto G, Tamburella F, Laurenza L, Foti C, Ditunno JF, Molinari M (2011). Validity and reliability of the 10-m walk test and the 6-min walk test in spinal cord injury patients. Spinal Cord.

[29] Kroll T, Kehn M, Ho PS, Groah S (2007). The SCI exercise self-efficacy scale (ESES): development and psychometric properties. Int J Behav Nutr Phys Act.

[30] Resnick B, Jenkins LS (2000). Testing the reliability and validity of the self-efficacy for exercise scale. Nurs Res.

[31] Sallis JF, Grossman RM, Pinski RB, Patterson TL, Nader PR (1987). The development of scales to measure social support for diet and exercise behaviors. Prev Med (Balt).

[32] Tulsky DS, Jette AM, Kisala PA, Kalpakjian C, Dijkers MP, Whiteneck G (2012). Spinal cord injury-functional index: item banks to measure physical functioning in individuals with spinal cord injury. Arch Phys Med Rehabil.

[33] Tulsky DS, Kisala PA, Victorson D, Tate DG, Heinemann AW, Charlifue S (2015). Overview of the spinal cord injury–quality of life (SCI-QOL) measurement system. J Spinal Cord Med.

[34] Hays RD, Morales LS (2001). The RAND-36 measure of health-related quality of life. Ann Med.

[35] Rosenthal R, Cooper H, Hedges L (1994). Parametric measures of effect size. Handb Res Synth.

[36] Pallant J. SPSS survival manual: a step by step guide to data analysis using IBM SPSS. Australian & New Zealand Journal of Public Health. 2011.

[37] Cohen J (1992). A power primer. Psychol Bull.

[38] Lai B, Cederberg K, Vanderbom KA, Bickel CS, Rimmer JH, Motl RW (2018). Characteristics of adults with neurologic disability recruited for exercise trials: a secondary analysis. Adapt Phys Act Q.

[39] Graham K, Yarar-Fisher C, Li J, McCully KM, Rimmer JH, Powell D (2019). Effects of high-intensity interval training versus moderate-intensity training on cardiometabolic health markers in individuals with spinal cord injury: a pilot study. Top Spinal Cord Inj Rehabil.

[40] Astorino TA, Thum JS (2018). Interval training elicits higher enjoyment versus moderate exercise in persons with spinal cord injury. J Spinal Cord Med.

[41] Thompson PD, Franklin BA, Balady GJ, Blair SN, Corrado D, Medicine AC of S (2007). Exercise and acute cardiovascular events: placing the risks into perspective: a scientific statement from the American Heart Association Council on Nutrition, Physical Activity, and Metabolism and the Council on Clinical Cardiology. Circulation.

[42] Rynecki ND, Siracuse BL, Ippolito JA, Beebe KS (2019). Injuries sustained during high intensity interval training: are modern fitness trends contributing to increased injury rates?. J Sports Med Phys Fit.

[43] Pelletier CA, Ditor DS, Latimer-Cheung AE, Warburton DE, Hicks AL (2014). Exercise equipment preferences among adults with spinal cord injury. Spinal Cord.

[44] Oka RK, King AC, Young DR (1995). Sources of social support as predictors of exercise adherence in women and men ages 50 to 65 years. Women’s Heal (Hillsdale, NJ).

[45] Pickett AC, Goldsmith A, Damon Z, Walker M (2016). The influence of sense of community on the perceived value of physical activity: a cross-context analysis. Leis Sci.

[46] Whiteman-Sandland J, Hawkins J, Clayton D (2018). The role of social capital and community belongingness for exercise adherence: An exploratory study of the CrossFit gym model. J Health Psychol.

[47] Kind AJH, Buckingham WR (2018). Making neighborhood-disadvantage metrics accessible—the neighborhood atlas. N Engl J Med.

[48] Health U of WS of M and P. Area Deprivation Index Version 3.2. [Internet]. [cited 2023 May 10]. Available from: https://www.neighborhoodatlas.medicine.wisc.edu/.

[49] Gill DP, Jones GR, Zou G, Speechley M (2012). Using a single question to assess physical activity in older adults: a reliability and validity study. BMC Med Res Methodol.

