# Correction: High intensity functional training for people with spinal cord injury & their care partners

**DOI:** 10.1038/s41393-024-01010-8

**Published:** 2024-06-20

**Authors:** Reed Handlery, Kaci Handlery, Dana Kahl, Lyndsie Koon, Elizabeth W. Regan

**Affiliations:** 1 Arkansas Colleges of Health Education, School of Physical Therapy, 7006 Chad Colley Blvd, Fort Smith, AR 72916 USA; 2 Research and Training Center on Independent Living, University of Kansas, Lawrence, KS USA; 3 University of South Carolina, Department of Exercise Science, Physical Therapy Program, Columbia, SC USA

**Keywords:** Public health, Spinal cord diseases

Correction to: *Spinal Cord* 10.1038/s41393-024-00977-8, published online 22 March 2024

In this article the statement in the Funding information section was incorrectly given as:

FUNDING: This work was supported in part through a Craig H. Neilsen Creating Opportunity & Independence grant (KH) and Seed Grant from the Arkansas Colleges of Health Education (RH). The funders played no role in the design, conduct, or reporting of this study.

and should read:

FUNDING: This work was supported in part through a Craig H. Neilsen Creating Opportunity & Independence grant (KH) and Seed Grant from the Arkansas Colleges of Health Education (RH). The funders played no role in the design, conduct, or reporting of this study. Publication fees for this article were supported by the Arkansas Colleges of Health Education Library Open Access Fund.

The original article has been corrected.

