# Supplementary material for: High intensity functional training for people with spinal cord injury & their care partners
Source: Spinal Cord. 2024 Mar 22;62(7):357–66. doi: 10.1038/s41393-024-00977-8 (PMC11230911; doi:10.1038/s41393-024-00977-8)
Supplement: Supplementary file 1 — Supplementary Material Legends [file 41393_2024_977_MOESM1_ESM.docx]

**Supplementary Material Legends**

**Appendix 1.** Content of 49 HIFT Exercise Sessions

Word Document

**Appendix 2.** Exercise Content with Adaptations & Primary Skill Focus

Excel Document
