# Supplementary material for: High intensity functional training for people with spinal cord injury & their care partners
Source: Spinal Cord. 2024 Mar 22;62(7):357–66. doi: 10.1038/s41393-024-00977-8 (PMC11230911; doi:10.1038/s41393-024-00977-8)
Supplement: Supplementary file 2 — Appendix 1 [file 41393_2024_977_MOESM2_ESM.docx]

Table of Contents

[Terminology 4](#_Toc144665073)

[Warmup 4](#_Toc144665074)

[Aerobic Warm Up: 4](#_Toc144665075)

[Shoulder Warm Up: 4](#_Toc144665076)

[Week 1 5](#_Toc144665077)

[Session 1 5](#_Toc144665078)

[Session 2 5](#_Toc144665079)

[Week 2 6](#_Toc144665080)

[Session 3 6](#_Toc144665081)

[Session 4 6](#_Toc144665082)

[Week 3 7](#_Toc144665083)

[Session 5 7](#_Toc144665084)

[Session 6 7](#_Toc144665085)

[Week 4 8](#_Toc144665086)

[Session 7 8](#_Toc144665087)

[Session 8 8](#_Toc144665088)

[Week 5 10](#_Toc144665089)

[Session 9 10](#_Toc144665090)

[Session 10 10](#_Toc144665091)

[Week 6 11](#_Toc144665092)

[Session 11 11](#_Toc144665093)

[Session 12 11](#_Toc144665094)

[Week 7 12](#_Toc144665095)

[Session 13 12](#_Toc144665096)

[Session 14 12](#_Toc144665097)

[Week 8 14](#_Toc144665098)

[Session 15 14](#_Toc144665099)

[Week 9 15](#_Toc144665100)

[Session 16 15](#_Toc144665101)

[Session 17 16](#_Toc144665102)

[Week 10 17](#_Toc144665103)

[Session 18 17](#_Toc144665104)

[Session 19 17](#_Toc144665105)

[Week 11 18](#_Toc144665106)

[Session 20 18](#_Toc144665107)

[Session 21 18](#_Toc144665108)

[Week 12 18](#_Toc144665109)

[Session 22 18](#_Toc144665110)

[Session 23 19](#_Toc144665111)

[Week 13 19](#_Toc144665112)

[Session 24 19](#_Toc144665113)

[Session 25 20](#_Toc144665114)

[Week 14 21](#_Toc144665115)

[Session 26 21](#_Toc144665116)

[Session 27 21](#_Toc144665117)

[Week 15 22](#_Toc144665118)

[Session 28 22](#_Toc144665119)

[Session 29 22](#_Toc144665120)

[Week 16 23](#_Toc144665121)

[Session 30 23](#_Toc144665122)

[Session 31 23](#_Toc144665123)

[Week 17 23](#_Toc144665124)

[Session 32 23](#_Toc144665125)

[Session 33 24](#_Toc144665126)

[Week 18 25](#_Toc144665127)

[Session 34 25](#_Toc144665128)

[Session 35 25](#_Toc144665129)

[Week 19 26](#_Toc144665130)

[Session 36 26](#_Toc144665131)

[Session 37 26](#_Toc144665132)

[Week 20 27](#_Toc144665133)

[Session 38 27](#_Toc144665134)

[Session 39 27](#_Toc144665135)

[Week 21 28](#_Toc144665136)

[Session 40 28](#_Toc144665137)

[Session 41 28](#_Toc144665138)

[Week 22 29](#_Toc144665139)

[Session 42 29](#_Toc144665140)

[Session 43 29](#_Toc144665141)

[Week 23 30](#_Toc144665142)

[Session 44 30](#_Toc144665143)

[Session 45 30](#_Toc144665144)

[Week 24 32](#_Toc144665145)

[Session 46 32](#_Toc144665146)

[Session 47 32](#_Toc144665147)

[Week 25 33](#_Toc144665148)

[Session 48 33](#_Toc144665149)

[Session 49 33](#_Toc144665150)

# Terminology

1:00 – One minute

:60 – 60 seconds

1:30 – One minute and 30 seconds

AMRAP – As Many Rounds (or repetitions) As Possible

EMOM – Every Minute (or 1.5, 2, etc.) On the Minute

MOVE – Participants chose their own form of movement for the prescribed exercise which could include skiing, biking, rowing, walking, or propelling

RPE – Rating of Perceived Exertion

# Warmup

## Aerobic Warm Up:

- 2-5 minute MOVE (SkiErg®, RowErg®, Bike, Ambulation, Wheelchair Propulsion)

Shoulder Warm Up: Eye level resistance banded exercises for One set, 10-15 repetitions, 1111 tempo

- Shoulder external rotation at 0 degrees abduction
- Shoulder internal rotation at 0 degrees abduction
- Reverse fly
- Dumbbell Scaption
- Dips/Press downs

# Week 1

## Session 1

Warmup

------------------------------------------------------------

AMRAP in 8 minutes

- 10 Banded pulldowns – pronated grip
- 50m move

Education

- Exercise safety, class schedule, intensity and challenge, rating of perceived exertion scale and daily assessment, instructor and participant introductions, sharing why participants joined

## Session 2

Warmup

------------------------------------------------------------

AMRAP in 8 minutes

- 10 medicine ball wall balls
- 5 medicine ball rotations (each direction)

--------------------5 Minute Rest---------------------

Intervals: 6 rounds, :20s work, 20s rest

- SkiErg® or RowErg® or Walk or Rope swing

# Week 2

## Session 3

Warmup

------------------------------------------------------------

EMOM: Every 2 minutes for 24 minutes

- 10-15 vertical press
- 10 medicine ball slams (forward or horizontal)
- 10-15 single arm banded row (each side)
- 5-10 u-turns

--------------------5 Minute Rest---------------------

Thunderstruck Intervals in 4:52:

- forward walking/wheelchair propulsion throughout “Thunderstruck”
- 1 burpee every time participant hears the word “thunder”

## Session 4

Warmup

------------------------------------------------------------

Partner Workout For Time (in pairs)

- 800–1200-meter total move (both partners exercising simultaneously)
- 50 lateral dead lifts + bent over row*
- 100-200 rope swings*
- 50 burpees*
- 800–1200-meter total move (both partners exercising simultaneously)

*Partner 1 exercises while partner 2 rests, alternating back and forth until total repetitions are completed

# Week 3

## Session 5

Warmup

------------------------------------------------------------

AMRAP in 20 Minutes

- 15-10-5 repetitions of the following:
  - Medicine ball slams (forward or horizontal)
  - Single arm banded press
  - Hoist
- Max distance move for remainder of time

--------------------5 Minute Rest---------------------

Mobility: Trunk

- 10 thoracic rotations (each side)*
- 10 thoracic extensions*
- 10 thoracic rotation + side bending (each side)*
- 10 overhead presses*
- 2 x 10s upper trapezius stretch (each side)
- *Using a PVC pipe

## Session 6

Warmup

------------------------------------------------------------

EMOM: Every 2 minutes for 16 minutes

- 10-15 banded pulldowns – pronated grip (2-second hold at bottom)
- 10-15 medicine ball wall crawl (2-second hold at top)

--------------------5 Minute Rest---------------------

Battle Rope Intervals: 8 rounds of :20s work, :30s rest

- Overhead static hold
- Drumroll
- Lateral raise
- Double slam
- Trunk twist

--------------------2 Minute Rest---------------------

- Drumroll
- Double slam
- Scaption raises
- Side swipes
- Drumroll

# Week 4

## Session 7

Warmup

------------------------------------------------------------

Shadow Boxing AMRAP with Gloves: :30s each of the following:

- Scaption
- Shoulder circles (forward)
- Shoulder circles (backward)
- Hip to sky
- Forearm circles (each direction)
- Overhead hold

Active Rest for 2 Minutes with Punches 1-6

- Jab and cross
- Jab and hook
- Jab and uppercut
- Jap, slip, hook
- Jab, cross, hook, uppercut

--------------------2 Minute Rest---------------------

Partner Heavy bag Intervals with Gloves: 1 round, 30s work, :30s rest

- Jab and cross
- Jab and hook
- Jab and uppercut
- Jap, slip, hook
- Jap, cross, hook, uppercut
- Head, body, head
- Jab, jab, hook
- Jab and cross pyramid) (2-4-6-8-10-8-6-4-2)

*Partners alternate work/rest (e.g., partner A works for :30s while partner B rests for :30s, then they switch).

--------------------2 Minute Rest---------------------

Partner Workout For Time: Rope Swings & U-turn pyramid

- 10 rope swings; 1 u-turn
- 20 rope swings; 2 u-turn
- 30 rope swings; 3 u-turn
- 40 rope swings; 4 u-turn
- 50 rope swings; 5 u-turn

*Partners alternate work/rest (e.g., partner A performs 10 rope swings while partner B rests, then partner B performs 1 u-turn while partner A rests. Once at the top of the pyramid, partners switch exercises and repeat the pyramid)

## Session 8

Warmup

------------------------------------------------------------

Group A: AMRAP in 10 minutes

- 25m forward sled drag
- 10-15 vertical press

--------------------5 Minute Rest---------------------

Group B: AMRAP in 10 minutes

- 8 medicine ball ground to shoulder
- 8 banded palloff press (each side)

--------------------3 Minute Rest---------------------

Mobility: Upper Extremities and Trunk, :30s of each of the following:

- Seated child’s pose
- Wrist flexor stretch (each side)
- Posterior shoulder stretch (each side)
- Shoulder rolls (forward)
- Shoulder rolls (backward)
- Seated latissimus dorsi stretch (each side)

# Week 5

## Session 9

Warm up

------------------------------------------------------------

AMRAP in 25 minutes

- 5-minute SkiErg®/RowErg®/Bike for max calories. Followed by
  - 50 battle rope drumrolls
  - 30 free weight side bends (15 each side)
  - 20 medicine ball rotations (10 each direction)
  - 10 medicine ball front raises

--------------------5 Minute Rest--------------------

Eye of the Tiger Move Intervals in 4:04

- Light intensity move during song verse
- High intensity move during song chorus

--------------------5 Minute Rest---------------------

Mobility: Upper Extremities and Trunk, :30s of each of the following:

- Seated child’s pose
- Posterior shoulder stretch (each side)
- Shoulder rolls (forward)
- Shoulder rolls (backward)
- Upper trapezius stretch (each side)

## Session 10

Warm up

------------------------------------------------------------

Partner Workout For Time (in teams of 3): 30-minute time cap

- 30 burpees*
- 60 hoists*
- 80 landmine press*
- 100-150 calorie SkiErg®/RowErg®/Bike
- 300 rope swings (150 forward, 150 backward)

* Two teammates may work while the other rests until total repetitions are complete. All teammates concurrently perform SkiErg®/RowErg®/Bike and rope swings

# Week 6

## Session 11

Warm up

------------------------------------------------------------

5-minute SkiErg®/RowErg®/Bike for max distance

--------------------5 Minute Rest---------------------

EMOM: Every 2 minutes for 18 minutes

- 16-20 medicine ball over shoulder wall taps
- 10-15 medicine ball wall crawls (2 second hold at top)
- 30-40 seal jacks

--------------------5 Minute Rest---------------------

Mobility: Upper Extremities and Trunk, :30s of each of the following:

- Neck circles (each direction)
- Posterior shoulder stretch (each side)
- Shoulder rolls (forward)
- Shoulder rolls (backward)
- Seated child’s pose (three angles)
- Wrist circles (each direction)

------------------------------------------------------------

Nutritional Education for 30 minutes

## Session 12

Warm up

------------------------------------------------------------

5-minute SkiErg®/RowErg®/Bike for max distance

--------------------5 Minute Rest---------------------

Group A: AMRAP in 8 minutes

- 25m forward sled drag
- 20 medicine ball rotations

--------------------5 Minute Rest---------------------

Group B: AMRAP in 8 minutes

- 10-15 vertical press
- 10-15 single arm banded row (each side)
- 5 burpees

--------------------5 Minute Rest---------------------

Cinco de Mayo Pinata Smash: Roll a die, perform the number of pushups and get that many attempts at the pinata for the number rolled

# Week 7

## Session 13

Warmup

------------------------------------------------------------

Group A: In 12 minutes

- Shadow Boxing AMRAP with Gloves: :30s each of the following:
  - Jab and cross
  - Jab and hook
  - Jab and upper cut
  - Jab, slip, hook
  - Jap, cross, hook, uppercut
- Heavy bag Intervals with Gloves: 30s work, :30s rest
  - Jab and cross
  - Jab and hook
  - Jap, cross, hook, uppercut
  - Head, body, head
  - Jab, jab, hook
  - Jab and cross pyramid (2-4-6-8-10-8-6-4-2)

--------------------3 Minute Rest---------------------

Group B: Intervals in 12 minutes

- Wheelchair users
  - Forward push-push-stop-burpee (4x 25m)
  - Backward push-push-stop-burpee (2x 25m)
  - Forward 1/4 way, turn, backward 1/4 of the way, repeat (4x 25m)
  - Backward 1/4 way, turn, forward 1/4 of the way, repeat (2x 25m)
  - Forward weave (2x 25m)
  - Backward weave (2x 25m)
- Ambulators
  - Forward step-step-step-stop-burpee (2x 25m)
  - Backwards step-step-step-stop (2x 25m)
  - Forward 1/4 way, turn, backward 1/4 way, repeat (2x 25m)
  - Backward 1/4 way, turn, forward 1/4 way, repeat (2x 25m)
  - Forward weave (2x 25m)
  - Backward weave (2x 25m)

------------------------------------------------------------

Mindfulness for 15 minutes

## Session 14

Warm Up

------------------------------------------------------------

EMOM: Every 2 minutes for 24 minutes

- :60s right landmine row
- :60s left landmine row
- 10 medicine ball slams (forward or horizontal)
- :60s dot drill (3-dots per person, instructor calls out a color, step or propel to dot)

--------------------5 Minute Rest---------------------

Rope Swing Intervals: 8 rounds, :20s work, 10s rest

# Week 8

## Session 15

Warm Up

------------------------------------------------------------

Team Workout For Time (entire class works together), “Family Ski Trip”

- SkiErg® 21,098 meters (half marathon distance) across 10 SkiErgometers (total)
- 350 medicine ball ground to shoulder (total)

# Week 9

## Session 16

Warm Up

------------------------------------------------------------

Free Weight Intervals: 6 rounds, :30s work, 30s rest

- I raise (bilateral)
- Y raise (bilateral)
- T raise (bilateral)
- Trunk rotations (each side, slow)
- 90/90 shoulder external rotation
- Lift and Chop (:15s each side)

--------------------2 Minute Rest---------------------

Battle Rope Intervals: 6 rounds of :30s work, :30s rest

- Drumroll
- Double slam
- Side swipe

--------------------2 Minute Rest---------------------

Battle Rope Intervals: 6 rounds of :20s work, :30s rest

- Drumroll
- Double slam
- Side swipe
- Outward circles
- Inward circles
- Snakes (in together, out apart)

--------------------2 Minute Rest---------------------

Battle Rope Intervals: 9 rounds of :10s work, :30s rest

- Drumroll
- Double slam
- Side swipe
- Outward circles
- Inward circles
- Snakes (in together, out apart)
- Jacks
- Trunk rotations (each direction, fast)
- Drumroll

--------------------2 Minute Rest---------------------

Mobility: Trunk

- 12 thoracic rotations (each side)*
- 12 thoracic extensions*
- 12 thoracic rotation + side bending (each side)*
- 12 scapula protraction and retraction
- 12 overhead presses*
- 12 overhead shoulder rotations (each side)

*Using a PVC pipe

## Session 17

Warm Up

------------------------------------------------------------

Group A. AMRAP in 14 minutes

- 30-20-10 repetitions of the following:
  - Burpees
  - Free weight side bends
  - Seal jacks
- Max distance sled forward sled drag

--------------------5 Minute Rest---------------------

Group B. AMRAP in 14 minutes

- 30-20-10 repetitions of the following:
  - SkiErg® for calories
  - Medicine ball over shoulder wall taps
- Max repetitions medicine ball wall ball

# Week 10

## Session 18

Warm Up

------------------------------------------------------------

Partner Workout For Time: “Murph”

- 1200/1600/2400-meter move (total)
- 50/75/100 hoists or pull ups or ring rows (total)
- 75/100/200 medicine ball wall balls or body weight squats (total)
- 1200/1600/2400-meter move (total)

## Session 19

Warm Up

------------------------------------------------------------

Mobility: Trunk

- 12 thoracic rotations (each side)*
- 12 thoracic extensions*
- 12 thoracic rotation + side bending (each side)*
- 12 scapula protraction and retraction
- 12 overhead presses*
- 12 overhead shoulder rotations (each side)

*Using a PVC pipe

------------------------------------------------------------

Postural Exercises

- - 6x10s chin tuck holds
  - 3x10s isometric cervical side bending holds (each side)
  - 15 band pull aparts (diagonal, each direction)
  - 10 banded serratus wall crawl
  - 15 I raise (bilateral)
  - 15 Y raise (bilateral)
  - 15 T raise (bilateral)
  - 5x5s seated dip holds

------------------------------------------------------------

5-minute move

------------------------------------------------------------

Mobility: Upper Extremities, 30s of each of the following:

- - Wrist flexion
  - Wrist extension
  - Radial deviation
  - Ulnar deviation
  - Pronation
  - Supination
  - Free weight shrugs

# Week 11

## Session 20

Warm Up

------------------------------------------------------------

EMOM: Every 2 minutes for 24 minutes

- 10 u-turns
- 15 band resisted ring dips (each side)
- 30 alternating ring chops
- :60s move

------------------------------------------------------------

Mindfulness for 15 minutes

## Session 21

Warm Up

------------------------------------------------------------

Group A. Ascending AMRAP in 16 minutes

- Side-facing sled pull (1, 2, 3, 4, 5, 6…)
- SkiErg® for calories (2, 4, 6, 8, 10, 12…)
- Medicine ball ground to shoulder (3, 6, 9, 12, 15, 18…)

--------------------5 Minute Rest---------------------

Group B. Barbell bench press 4x5-8 repetitions

# Week 12

## Session 22

Warm Up

------------------------------------------------------------

5-minute SkiErg®/RowErg®/Bike for max distance

--------------------5 Minute Rest---------------------

Partner Workout For Time: “Flag Day”

- - 50 seal jacks
  - 50 burpees
  - 50 bilateral weighted bicep curls
  - 50 free weight side bends (each side)
  - 50 band pull aparts
  - 50 plate wheelies
  - 50 u-turns
  - 50m sled pull
  - 50 medicine ball rotational toss (each side)
  - 50 medicine ball ground to over shoulder
  - 50 medicine ball wall balls
  - 50 calorie SkiErg®/RowErg®/Bike
  - 50 lengths of the floor (25m) ambulation or propulsion

## Session 23

Warm Up

------------------------------------------------------------

Mobility: Trunk

- 12 thoracic rotations (each side)*
- 12 thoracic extensions*
- 12 thoracic rotation + side bending (each side)*
- 12 scapula protraction and retraction
- 12 overhead presses*
- 12 overhead shoulder rotations (each side)
- *Using a PVC pipe

------------------------------------------------------------

EMOM: Every 90 seconds for 18 minutes

- - :45s landmine press
  - :45s banded pull downs – supinated grip
  - :30s banded palloff press (each side)
  - :45s medicine ball rotations (each direction)

--------------------5 Minute Rest---------------------

Team Relay For Time: “You Push, I’ll Pull”

- Teams alternate between:
  - 250m SkiErg®
  - 250m Ambulation/Propulsion
  - Final 2 athletes perform 3 rope climbs followed by 250m SkiErg®

# Week 13

## Session 24

Warm Up

------------------------------------------------------------

Team Workout For Time (entire class works together), “Summer Solstice”

- Perform 1,432 repetitions (total) of any of the following (50-minute time cap)
  - Bodyweight
    - Burpees
    - Seal jacks
    - Seated dips (3 second hold)
    - Jab-cross-hook-upper cut (each sequence = 1 repetition)
    - U - turns
    - Ring rows
    - Squats
    - Reverse lunge
    - Double unders
  - Medicine ball
    - Wall ball
    - Rotations (both sides = 1 repetition)
    - Slams (forward or horizontal)
    - Over shoulder wall taps
    - Ground to shoulder
    - Push & get (one floor length (25m) = 10 repetitions)
  - Free Weight
    - Vertical press
    - Side bend
    - Bent over row
  - Rope
    - Hoist
    - Forward sled drag (one floor length (25m) = 10 repetitions)
    - Rope swings (10 contacts = 1 repetition)
  - MOVE
    - SkiErg®/RowErg®/Bike (1 calorie = 1 repetition)
    - Walk (one floor length (25m) = 1 repetition)
    - Propulsion (one floor length (25m) = 1 repetition)

## Session 25

Warm Up

------------------------------------------------------------

5-minute SkiErg®/RowErg®/Bike for max distance

--------------------5 Minute Rest---------------------

Strength Training: Barbell bench press 3x8-10 repetitions

--------------------3 Minute Rest---------------------

Battle Rope Intervals: 2x8 rounds of :20s work, :10s rest; 2 minutes rest between rounds

- Drumroll
- Snakes (in together, out apart)
- Circles (inward, outward)

--------------------5 Minute Rest---------------------

AMRAP in 10 minutes

- 5 medicine ball slams (forward or horizontal)
- 10 medicine ball wall balls
- 15 medicine ball ground to shoulder
- 20 medicine ball rotations (each direction)

--------------------3 Minute Rest---------------------

Thunderstruck Intervals in 4:52:

- forward walking/wheelchair propulsion throughout “Thunderstruck”
- 1 burpee every time participant hears the word “thunder”

# Week 14

## Session 26

Warmup

------------------------------------------------------------

AMRAP in 8 minutes

- 10 medicine ball wall balls
- 10 pallof press (each side)

--------------------5 Minute Rest---------------------

AMRAP in 8 minutes

- 5 medicine ball slams (forward or horizontal)
- 10 medicine ball rotations (each side)

--------------------5 Minute Rest---------------------

Rope Swing Intervals: 8 rounds of :20s work, 20s rest

## Session 27

Warmup

------------------------------------------------------------

Partner Workout AMRAP with 25-minute time cap: Complete 10-20-30-40-50…etc. of the following:

- Hoist and hold
- Free weight side bends
- Free weight curl to press
- SkiErg®/RowErg®/Bike calories or ambulation/propulsion

*Divide repetitions between partners as needed

--------------------5 Minute Rest---------------------

Team Burpee Pyramid For Time: Perform the following ascending repetition scheme alternating between teams (Team A performs 1 rep, Team B performs 1 rep; team A performs 2 reps; Team B performs 2 reps, etc.)

- 1-2-3-4-5-6-7-8-7-6-5-4-3-2-1

# Week 15

## Session 28

Warmup

------------------------------------------------------------

EMOM: Every 2 minutes for 18 minutes

- 15 rope tricep push downs (3s hold at bottom)
- 15 free weight bent over row (each side)
- 5-10 u-turns

--------------------5 Minute Rest---------------------

Reverse Sled Drag AMRAP in 10 minutes

- increase weight every other floor length (25m) until time reached

--------------------5 Minute Rest---------------------

4-minute move

## Session 29

Warmup

------------------------------------------------------------

Group A. “Andrea” For Time

- 50 barbell bench press
- 50 medicine ball rotations (total)
- 50 medicine ball ground to shoulder
- 500m SkiErg®

--------------------5 Minute Rest---------------------

Sugar, Sugar Intervals for 2:48

- Seal jacks throughout “Sugar, Sugar”
- 1 burpee every time participant hears the word “sugar,” “honey,” “sweet,” etc.

# Week 16

## Session 30

Warmup

------------------------------------------------------------

Partner Workout AMRAP in 10 minuets

- 10 medicine ball wall balls
- 15 medicine ball rotational toss (switch sides each round)
- 30 seal jacks

--------------------5 Minute Rest---------------------

Partner Workout AMRAP in 10 minuets

- 10 landmine press
- 15 band pull aparts
- 30 battle rope drumroll (60 contacts total)

--------------------5 Minute Rest---------------------

Move Intervals

- 1:1, easy:hard effort: 10s:10s, 20s:20s, 30s:30s, 40s:40s, 50s:50s, 60s:60s: 50s:50s: 60s:60s

## Session 31

Warmup

------------------------------------------------------------

Group A. “Chelsea” For Rounds For Time (12-minute time cap)

- 12-meter sled pull
- 12 burpees
- 12-meter reverse sled drag

Max rope swing contacts for remainder of time

Group B. Mobility: Trunk and Upper Extremities

- 20 thoracic rotation (each side)
- 20 thoracic extension
- 20 snow angels
- :30s seated child’s pose
- :30s posterior shoulder stretch (each side)
- :30s shoulder rolls forward
- :30s shoulder rolls backward
- :30s forearm circles (each side)

--------------------5 Minute Rest---------------------

Partner SkiErg®/RowErg®/Bike Pyramid AMRAP in 8 minutes

- Both partners SkiErg®/RowErg®/Bike at a light intensity and alternate bursts of high intensity at a 1:1 ratio, ascending by 5 seconds (bike), or 1 stroke (SkiErg®/RowErg®) until reaching 10 seconds or strokes, then descending back down

# Week 17

## Session 32

Warmup

------------------------------------------------------------

Partner Workout

- 5-minute SkiErg®/RowErg®/Bike (total)
- AMRAP in 20 minutes
  - 5 partner hoists (total)
  - 10 medicine ball wall balls (total)
  - 15 medicine ball rotations (each direction (total)
- 5-minute SkiErg®/RowErg®/Bike (total)

--------------------5 Minute Rest---------------------

Mobility: Trunk and Upper Extremities

- 20 cervical flexion and extension
- 20 cervical rotation
- 20 thoracic extension
- :30s seated child’s pose
- :30s shoulder rolls - forward
- :30s shoulder rolls backward
- :30s forearm circles (each side)

## Session 33

Warmup

------------------------------------------------------------

Free Weight Intervals: 6 rounds, :30s work, 30s rest

- I raise (bilateral)
- Y raise (bilateral)
- T raise (bilateral)
- Trunk rotations (each side, slow)
- 90/90 shoulder external rotation
- Lift and Chop (:15s each side)

--------------------5 Minute Rest---------------------

EMOM: Every 2 minutes for 24 minutes

- :60s bob and weave
- :60s single arm banded row with 3 eccentric stops right
- :60s single arm banded row with 3 eccentric stops left
- :60s medicine ball ground to up and over box

--------------------5 Minute Rest---------------------

Move Intervals: 6 rounds, :20s work, :10s rest

------------------------------------------------------------

Cooldown: 1 minute move

# Week 18

## Session 34

Warmup

------------------------------------------------------------

EMOM: Every 2 minutes for 24 minutes

- :60s pullups
- :60s good morning to overhead press
- :60s landmine twist
- :60s dot drill (3-dots per participant; instructor calls out a dot color and participant propels forward to that color)

--------------------5 Minute Rest---------------------

Team Relay For Time: “You Push, I’ll Pull”

- Teams alternate between:
  - 250m SkiErg®
  - 100m Ambulation/Propulsion

## Session 35

Warmup

------------------------------------------------------------

Team Workout For Time (entire class works together), “Family Ski Trip”

- SkiErg® 21,098 meters (half marathon distance) across 10 SkiErgometers (total)
- 450 medicine ball ground to shoulder (total)

--------------------5 Minute Rest---------------------

Bilateral Free Weight Bicep Curl Pyramid: 1:1 work:rest ratio; ascending from 1 to 10 repetitions then back down to 1 repetition

# Week 19

## Session 36

Warmup

------------------------------------------------------------

Partner Workout Rope Swing Intervals for 6 minutes

- 1:1 work:rest ratio, ascending from 10 to 100 contacts then back down to 10

--------------------5 Minute Rest---------------------

EMOM: Every 90 seconds for 18 minutes

- 15 medicine ball lateral wall balls (right)
- 15 medicine ball lateral wall balls (left)
- 10 medicine ball slams (forward or horiztonal)
- :45s overhead medicine ball hold

--------------------5 Minute Rest---------------------

Partner Workout Hoist and Hold Pyramid AMRAP in 5 minutes

- Partner A isometrically holds weighted rope while Partner B performs hoist repetitions (descending from 10 repetitions to 1 repetition) with partners switching back and forth each repetition scheme

## Session 37

Warmup

------------------------------------------------------------

Strength Training: Bench, Balls, and Burpees

- Barbell bench press 4x10
- Medicine ball overhead backward toss 4x10
- Burpees 4x10

--------------------5 Minute Rest---------------------

Partner Workout: Ski and Sled AMRAP in 12 minutes

- Partner A SkiErg® for calories while Partner B forward sled drag 40m
- Partners switch when sled drags are complete

# Week 20

## Session 38

Warmup

------------------------------------------------------------

Battle Rope Intervals: 3x6 rounds of :20s work, :10s rest; 2 minutes rest between rounds

- Drumroll
- Double slam
- Side swipe
- Circles (inward, outward)
- Snakes (in together, out apart)
- Drumroll

--------------------5 Minute Rest---------------------

EMOM: Every 2 minutes for 24 minutes

- :60s banded pull downs – pronated grip
- :60s banded pallof press
- :60 plate wheelies + burpees
- :60s u-turns

## Session 39

Warmup

------------------------------------------------------------

Free Weight Intervals: 2x6 rounds of :20s work, :20s rest; 2 minutes rest between rounds

- rope swing forward
- rope swing backward
- Y raise (bilateral)
- T raise (bilateral)
- rope swing backward
- rope swing forward

--------------------5 Minute Rest---------------------

Group A. “Andrea” For Time

- 50 barbell bench press
- 50 medicine ball rotations (total)
- 50 medicine ball ground to shoulder
- 500m SkiErg®

Group B. 3-minute SkiErg®

--------------------5 Minute Rest---------------------

Thunderstruck Intervals in 4:52:

- forward walking/wheelchair propulsion throughout “Thunderstruck”
- 1 burpee every time participant hears the word “thunder”

# Week 21

## Session 40

Warmup

------------------------------------------------------------

Group A. AMRAP in 14 minuets

- 30-20-10 of each the following:
  - Burpees
  - Free weight side bends (each side)
  - Seal jacks
- Max distance reverse sled drag

--------------------5 Minute Rest---------------------

Group B. AMRAP in 10 minutes, “Carol”

- 3-7-17 of each of the following:
  - SkiErg® calories
  - Medicine ball head taps
- Max medicine ball wall ball repetitions for remainder of time

## Session 41

Warmup

------------------------------------------------------------

Move Intervals: 2x4 minute move with :90s rest between rounds

--------------------3 Minute Rest---------------------

EMOM: Every 60 seconds for 9 minutes

- :40s medicine ball over shoulder wall taps
- :40s medicine ball rotations (each direction
- :30s medicine ball front raise hold

--------------------3 Minute Rest---------------------

Rope Intervals: 2x6 rounds, :30s work, 30s rest; :60s rest between rounds

- Hoist
- Hoist
- Rope Tricep pushdown
- Rope Tricep pushdown
- Hoist + hold
- Hoist + hold

# Week 22

## Session 42

Warmup

------------------------------------------------------------

Group A. “Chelsea” For Rounds For Time (12-minute time cap)

- 12-meter sled pull
- 12 burpees
- 12-meter reverse sled drag

Max rope swing contacts for remainder of time

--------------------3 Minute Rest---------------------

Group B. SkiErg® Intervals for 12 minutes

- 2 minutes easy
- :15s work; 15s rest
- :30s work; 30 rest
- :45s work; 55s rest
- :60s work; 60s rest
- :45s work; 45s rest
- :30s work;30s rest
- :15s work; 15s rest
- 2 minutes easy

--------------------3 Minute Rest---------------------

Team Relay For Time: “You Turn, I’ll Pull”

- Teams with pairs alternate between:
  - 100m SkiErg®
  - 10 u-turns

## Session 43

Warmup

------------------------------------------------------------

Partner Workout AMRAP in 33 minutes

- 9 hoists (each)
- 29 landmine presses (total)
- 89 calorie SkiErg®/RowErg®/Bike (total)

--------------------5 Minute Rest---------------------

Sugar, Sugar Intervals for 2:48

- Medicine ball rotations (each side) throughout “Sugar, Sugar”
- 1 medicine ball wall ball every time participant hears the word “sugar,” “honey,” “sweet,” etc.

# Week 23

## Session 44

Warmup

------------------------------------------------------------

Partner Workout AMRAP in 6 minutes

- Alternating sled pull

--------------------3 Minute Rest---------------------

Group A. In 15 minutes

- Shadow Boxing AMRAP with Gloves: :30s each of the following in 15 minutes
  - Jab and cross
  - Jab and hook
  - Jab and uppercut
  - Jap, slip, hook
  - Jab, cross, hook, uppercut
- Partner Heavy bag Intervals with Gloves: 1 round, 30s work, :30s rest
  - Jab and cross
  - Jab and hook
  - Jap, cross, hook, uppercut
  - Head, body, head
  - Jab, jab, hook
  - Jab and cross pyramid) (2-4-6-8-10-8-6-4-2)

--------------------3 Minute Rest---------------------

Group B: Intervals in 15 minutes

- Wheelchair users
  - Forward push-push-stop-burpee (4x 25m)
  - Backward push-push-stop-burpee (2x 25m)
  - Forward 1/4 way, turn, backward 1/4 of the way, repeat (4x 25m)
  - Backward 1/4 way, turn, forward 1/4 of the way, repeat (2x 25m)
  - Forward weave (2x 25m)
  - Backward weave (2x 25m)
- Ambulators
  - Forward step-step-step-stop-burpee (2x 25m)
  - Backwards step-step-step-stop (2x 25m)
  - Forward 1/4 way, turn, backward 1/4 way, repeat (2x 25m)
  - Backward 1/4 way, turn, forward 1/4 way, repeat (2x 25m)
  - Forward weave (2x 25m)
  - Backward weave (2x 25m)

--------------------3 Minute Rest---------------------

Partner SkiErg®/RowErg®/Bike Pyramid AMRAP in 8 minutes

- Both partners SkiErg®/RowErg®/Bike at a light intensity and alternate bursts of high intensity at a 1:1 ratio, ascending by 5 seconds (bike), or 1 stroke (SkiErg®/RowErg®) until reaching 10 seconds or strokes, then descending back down

## Session 45

Warmup

------------------------------------------------------------

Group A. Hammer Intervals: 2x8 rounds, :20s work, 20s rest; 2 minutes rest between rounds

- hammer over shoulder slam
- hammer over shoulder slam
- hammer low hit
- hammer low hit
- hammer ditch digger
- hammer ditch digger
- hammer hold
- hammer hold

--------------------3 Minute Rest---------------------

Group B. AMRAP x 12 minutes

- 12 free weight dynamic curl to press
- 12 free weight side bends (each side)
- 12 calorie SkiErg®/RowErg®/Bike

--------------------3 Minute Rest---------------------

Partner Workout Ski and Skip AMRAP in 10 minutes

- Partner A SkiErg®/RowErg®/Bike 10-15 calories while Partner B rope swings for maximum reps until Partner A completes the prescribed repetitions

# Week 24

## Session 46

Warmup

------------------------------------------------------------

Partner Workout Medicine Ball AMRAP in 12 minutes

Partner A

- 10 medicine ball wall balls
- 10 medicine ball burpees
- 20 medicine ball rotational lifts (10 each side)
- 20 medicine ball rotations (10 each direction)

Partner B moves until Partner A completes the prescribed repetitions

--------------------3 Minute Rest---------------------

Strength Training: Bench, Row, & Slam

- Barbell bench press 3x10-12
- Landmine row 3x10-12 (each side)
- Medicine ball slam (3-way) 3x10

--------------------3 Minute Rest---------------------

Rope Swing Intervals: 8 rounds of :20s work, 10s rest

## Session 47

Warmup

------------------------------------------------------------

Battle Rope Intervals: 3x6 rounds, :20s work, 10s rest; 90 seconds rest between rounds

- Drumroll
- Double slam
- Side swipe
- Circles (inward, outward)
- Snakes (in together, out apart)
- Drumroll

--------------------3 Minute Rest---------------------

AMRAP in 14 minutes

- 10-20-30 of repetitions of the following:
  - bob & weave burpee
  - Ring rows
  - u-turns
- Max calories SkiErg®/RowErg®/Bike for remainder of time

-------------------3 Minute Rest--------------------

House of Cards for 8 minutes

- Cards drawn randomly from card deck. Participants perform the number of repetitions based on the number on the drawn card. Exercises as follows:
  - Hearts – burpees
  - Diamonds – free weight curl to press
  - Spades – seal jacks
  - Clubs – medicine ball slams (forward or horizonal)

# Week 25

## Session 48

Warmup

------------------------------------------------------------

8-minute move – goal to negative split

-------------------3 Minute Rest--------------------

Free Weight Intervals: 6 rounds, :40s work, 20s rest

- I raise (bilateral)
- Y raise (bilateral)
- T raise (bilateral)
- Trunk rotations (each side, slow)
- 90/90 shoulder external rotation
- Lift and Chop (:15s each side)

--------------------3 Minute Rest---------------------

Partner Workout: Ski and Sled AMRAP in 15 minutes

- Partner A SkiErg® for calories while Partner B forward sled drag 40m
- Partners switch when sled drags are complete

## Session 49

Warmup

------------------------------------------------------------

Partner SkiErg®/RowErg®/Bike Pyramid AMRAP in 8 minutes

- Both partners SkiErg®/RowErg®/Bike at a light intensity and alternate bursts of high intensity at a 1:1 ratio, ascending by 5 seconds (bike), or 1 stroke (SkiErg®/RowErg®) until reaching 10 seconds or strokes, then descending back down

--------------------3 Minute Rest---------------------

EMOM: Every 90 seconds for 18 minutes

- :60s hoist
- :60 u-turns
- :30s medicine ball burpee
- 10 medicine ball slams (3-way)

Partner Workout: Rope Swing Pyramid AMRAP in 6 minutes

- 1:1 work:rest ratio; ascending from 10 to 100 repetitions then back down to 10 repetitions

--------------------3 Minute Rest---------------------

Team Relay For Time:

- Teams alternate between 100m SkiErg®, Wheelie+Burpees, forward sled drag for 20m
